# Natural Polymers for the Maintenance of Oral Health: Review of Recent Advances and Perspectives

**DOI:** 10.3390/ijms221910337

**Published:** 2021-09-25

**Authors:** Anna Paradowska-Stolarz, Mieszko Wieckiewicz, Artur Owczarek, Joanna Wezgowiec

**Affiliations:** 1 Division of Dentofacial Anomalies, Department of Maxillofacial Orthopedics and Orthodontics, Wroclaw Medical University, 50-425 Wroclaw, Poland; anna.paradowska-stolarz@umw.edu.pl; 2 Department of Experimental Dentistry, Wroclaw Medical University, 50-425 Wroclaw, Poland; joanna.wezgowiec@umw.edu.pl; 3 Department of Drug Form Technology, Wroclaw Medical University, 50-556 Wroclaw, Poland; artur.owczarek@umw.edu.pl

**Keywords:** polymers, biopolymers, dentistry, biomaterials, tissue engineering, drug delivery, collagen, chitosan

## Abstract

The success of modern dental treatment is strongly dependent on the materials used both temporarily and permanently. Among all dental materials, polymers are a very important class with a wide spectrum of applications. This review aims to provide a state-of-the-art overview of the recent advances in the field of natural polymers used to maintain or restore oral health. It focuses on the properties of the most common proteins and polysaccharides of natural origin in terms of meeting the specific biological requirements in the increasingly demanding field of modern dentistry. The use of naturally derived polymers in different dental specialties for preventive and therapeutic purposes has been discussed. The major fields of application cover caries and the management of periodontal diseases, the fabrication of membranes and scaffolds for the regeneration of dental structures, the manufacturing of oral appliances and dentures as well as providing systems for oral drug delivery. This paper also includes a comparative characteristic of natural and synthetic dental polymers. Finally, the current review highlights new perspectives, possible future advancements, as well as challenges that may be encountered by researchers in the field of dental applications of polymers of natural origin.

## 1. Introduction

Due to the remarkable technological development of material science that occurred in the past 30 years, both natural and synthetic biomaterials are now an indispensable part of modern medicine. They could be used to repair or replace defective organs [1]. Due to their biocompatibility, they can cause the specific response of the body, not affecting the immune system. Apart from a wide range of applications in various medical specialties, biomaterials are also commonly used in dentistry for restoring the masticatory system and improving oral health [2,3].

The main groups of dental materials are polymers, ceramics, metal alloys and composites. Among them, polymers are particularly versatile and multipurpose [4]. The polymeric materials most commonly used in dentistry include poly(methyl methacrylate) (PMMA), vulcanite, celluloid, phenol formaldehyde and polyvinyl chloride (PVC). They are used for manufacturing complete and partial dentures and could be added to denture soft liners, sealants and resin cements [4,5,6].

In general, polymers can be divided into synthetic (e.g., polyolefines, fluorinated polymers, polyesters, silicones) and natural polymers (e.g., polysaccharides and proteins) [7,8]. Synthetic polymers are very commonly present in items of everyday use, as they are found in plastic, rubber, fibers, adhesives, plexiglasses, composites and thermosets. They are among the best-selling materials in global sales (USD 500 billion annually) [4]. Polymers of natural origin (e.g., shellac, natural rubber, silk, amber, wool, etc.) have been used for different purposes for centuries [1,4,9].

From a medical point of view, materials based on natural polymers have many beneficial properties, such as biocompatibility, good availability and biodegradability, which is the ability to be degraded in biological systems by the hydrolysis of bonds along the polymer chain followed by oxidation. Therefore, many natural polymers can be decomposed to carbon dioxide, methane, water and biomass by microorganisms or through other biological processes, dependent on several factors, i.e., polymer structure, polymer morphology and molecular weight [10,11]. Another reason to consider natural polymers is the low cost of obtaining and producing them. For these reasons, they have attracted the attention of many research groups [5,12]. The biocompatibility and functional properties of polymers for oral applications are among the most important clinical issues. When choosing a polymer, one should consider its safety for all oral tissues—gums, mucosa, pulp and bone—additionally eliminating the risk of leaching or the diffusion of toxic components from materials. Such diffused components could be subsequently absorbed into the body, causing systemic reactions, including toxicity, tissue damage, teratogenic or carcinogenic effects, as well as inducing allergic reactions. Additionally, the effect of dental polymers on blood vessels should also be taken into consideration because the oral cavity is highly vascularized. Several reports from experimental studies provided evidence of a pronounced vasodilator effect of dental polymers that impair pulp healing by promoting hemorrhage [6,13].

This study aimed to discuss the potential of natural polymers in various dental applications. To the best of our knowledge, this is the first review article concerning dental applications of natural polymers; therefore, a comprehensive and critical presentation of the recent advances and future perspectives of natural polymers applied for oral health maintenance has been offered.

## 2. Material and Methods

**Figure 1:**
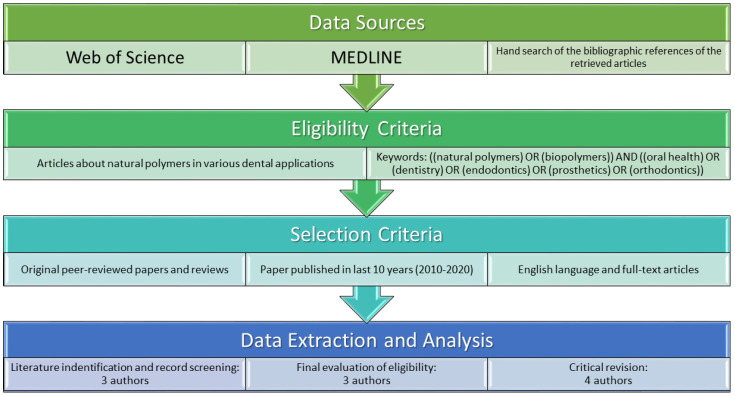
Flow diagram presenting the search strategy.

The PubMed/MEDLINE and Web of Science databases were searched to obtain up-to-date information on the topic of dental polymers of natural origin. The articles taken into account were original peer-reviewed papers and reviews published in English in the last 10 years. The authors excluded papers without clear scientific background and outstanding practical aspects. Figure 1 presents the strategy of the literature research.

## 3. Chemical Classification and Biological Activity of Natural Dental Polymers

**Figure 2:**
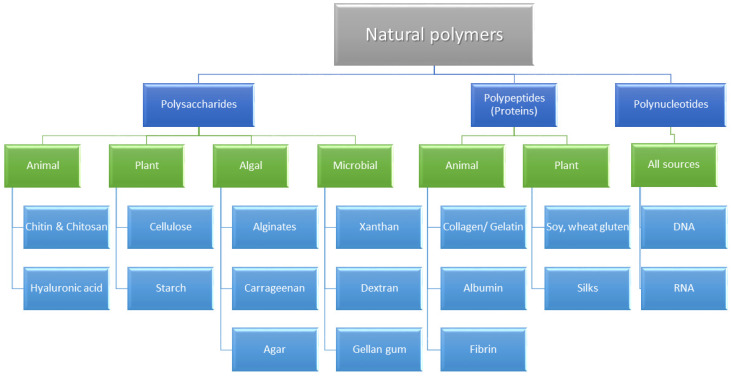
Classification of natural polymers based on their chemical structure and their source of origin [14].

An overview of the most important natural polymers classified as polysaccharides, proteins or polynucleotides is presented below. Additionally, a classification of natural polymers based on their chemical structure and their source of origin is summarized in Figure 2.

### 3.1. Polysaccharides

Polysaccharides are polymeric carbohydrates [(CH_2_O)_n_] composed of monosaccharides that are bound together by glycosidic linkages. Their structure might be linear or branched. They are very commonly found in living organisms, either as storage polysaccharides (e.g., starch and glycogen) or as structural polysaccharides (cellulose, chitin, pectins) [13,14,15,16]. Bacterial polysaccharides form a shield around bacterial molecules to protect bacteria from the immune system. A mixture of those polysaccharides is used for the production of vaccines [17,18].

In dentistry, the most commonly used and explored polysaccharides are chitosan, cellulose, hyaluronic acid, alginates and agarose.

#### 3.1.1. Chitosan

Chitosan is a natural cationic polysaccharide, derived from chitin—a structural polymer present in the shells of crustaceans, such as lobsters, crabs and shrimps. It has antifungal and antimicrobial activity against *Streptococcus mutans* and could have a hemostatic effect. It is also successfully used for tissue engineering due to its biocompatibility, biodegradation, osteoconductivity and affinity for biomolecules [19]. Chitosan sponge with interfering mRNA has great potential for being used in bone regeneration—it has been proven that such a system not only promotes osteogenesis but also helps with angiogenesis [20,21]. The biocompatibility of chitosan was investigated by many research groups [22,23,24,25]. They observed early neutrophil migration to the implantation area. This phenomenon resolved with increasing post-implantation time, whereby no other symptoms of inflammation, such as erythema or edema, were recorded. In addition, a very low incidence of specific immune reactions was observed. The formation of normal granulation tissue associated with accelerated angiogenesis occurs as a typical healing response. The results of the conducted research confirmed that chitosan and chitosan-based materials are characterized by a high degree of deacetylation and biocompatibility. In addition, the attachment of bioactive molecules to biocomposite scaffolds obtained on the basis of chitosan can not only promote the proliferation and differentiation of stem cells and accelerate tissue regeneration, but also induce angiogenesis and vascularization in various animal models, with a perspective for human application [26].

#### 3.1.2. Cellulose

Cellulose is a high molecular weight polysaccharide that is present in plant cell walls, responsible for the structural integrity and protection of plants [19]. It could also be produced by microorganisms, such materials are named bacterial cellulose and have superior physicochemical properties when compared to plant cellulose. Both plant and bacterial cellulose have great potential for application in the biomedical area, including dentistry [27]. As an important structural polysaccharide, cellulose is particularly useful as a component of drug delivery systems and scaffold for wound dressing [28].

#### 3.1.3. Hyaluronic Acid

Hyaluronic acid (HA) is a glycosaminoglycan composed of repeating units of N-acetyl-D-glucosamine and D-glucuronic acid. This linear polysaccharide is widely present in human cells. It is the main substance of the extracellular matrix of human skin, connective tissue, synovial fluid and other tissues, characterized by high biocompatibility, ease of chemical functionalization, biodegradability, hydrophilicity and non-immunogenicity. HA has the ability to maintain a humid environment. It is widely used in biomedical applications such as surgery (plastic, ocular, osteoarthritis), drug delivery and tissue engineering. Due to its ability to bond water molecules, it plays a key role in wound healing and tissue repair. It is also widely used in aesthetic dentistry to restore tissue volume. Since it is a strong antioxidant, it stimulates growth factors, cellular constituents and the migration of cells. In dentistry, HA could be used to improve wound healing and the restoration of periodontal tissue volume after dental procedures. It was revealed that hydrogel formulation containing polynucleotides and hyaluronic acid could enhance wound healing, as it promoted cell growth and viability and supported the formation of dense multilayered cell nodules in an in vitro model of gingival fibroblasts [29]. Clinical studies show that beneficial effects of HA application were also observed in patients with chronic inflammatory gingival and periodontal disease and in patients with oral ulcers [30].

#### 3.1.4. Sodium Alginate

Alginate is a linear anionic polysaccharide, a derivative of alginic acid consisting of 1,4-linked d-mannuronate residues and 1,4-linked l-guluronates. It could be extracted from different sources of algae with different compositions. The degree of biocompatibility of alginate depends on its composition, in particular the ratio of poly α-L-guluronic acid and β-D-mannuronic acid blocks, and the purity level. Polymers containing higher levels of β-D-mannuronic acid blocks are less immunogenic than alginates with a higher level of poly α-L-guluronic acid blocks [31]. Due to its advantageous properties, such as biocompatibility, biodegradability, mucoadhesion and low-cost production, sodium alginate has multiple medical applications, including drug and gene delivery, tissue engineering and wound healing. However, it also has some limitations, such as poor mechanical properties, hydrophilicity and microbial degradation, which could be overcome by combining it with other polymers [32]. Additionally, one of the most common application of alginates in dentistry is connected with their use for taking oral impressions. Alginate impressions are a necessary stage of treatment planning that enables the manufacture of orthodontic appliances, dentures and intraoral splints matched to the morphology of oral tissues. However, due to the various limitations of impression materials, in modern digital dentistry, they are more often replaced with intraoral scans [33,34].

#### 3.1.5. Agarose

Agarose is a polysaccharide containing repeating units of agarobiose, a disaccharide of D-galactose and 3,6-anhydro-1galactopyranose. Due to its biocompatibility, it could be a suitable medium for cell encapsulation. On the other hand, it is nondegradable in the human body because of the absence of an appropriate enzyme [35].

In dentistry, agarose is used for biomimetic remineralization, which is possible on the basis of a durable matrix for crystal formation. Agarose hydrogel is able to control the size and form of the hydroxyapatite crystal. It acts as a matrix for enamel rebuilding through interaction between the hydroxyl group of agarose and calcium, as well as providing a mineral reservoir for further remineralization. That reaction leads to the conclusion that agarose hydrogel has the potential for treating early carious lesions [36]. Other findings show that agarose gel may be used as a scaffold for bone regeneration, especially when combined with hydroxyapatite (HA) or calcium carbonate (CaCO_3_) [37,38].

#### 3.1.6. Synthetic Polysaccharides Based on Monomers Obtained from Natural Resources

Some other polymers synthesized from monomers of natural origin may also play a crucial role in hard tissue engineering and remodeling. Among them, the most commonly used are poly(α-hydroxy esters), including poly(lactic acid) (PLA) and poly(glycolic acid) (PGA), as well as poly(lactic-co-glycolide) (PLGA) copolymers. PLGA seems to be a particularly attractive material for that purpose. It is biocompatible, has been approved for clinical use in humans, has the potential to provide interaction with other biological materials and is suitable to be used by people who find animal-derived products objectionable. PLGA is used in a large variety of forms, such as porous scaffolds, hydrogels, films or microspheres [39].

### 3.2. Proteins

Proteins are built of amino acid monomers. They contain 50 or more amino acids, which distinguishes them from peptides. The main chemical elements found in proteins are C, N, O, H and S, sometimes P or ions of metals. Amino acids are joined by peptide bonds that are a type of a covalent bond between the carboxyl group of one amino acid and the amino group of the other amino acid [40]. It has been proven that materials based on proteins, due to their high biocompatibility and high affinity with other matrix proteins, are excellent systems for the production of bone grafts [41].

#### 3.2.1. Collagen

In nature, collagen is one of the most abundant cellular matrix proteins [42]. It is present in the gingival epithelium. It is widely applied in the restoration of skin-like structures. In dentistry, it is used mainly to facilitate the process of healing. It was revealed that the first stage of healing after the extraction is similar regardless of adding collagen to the socket, but the quality of bone after 4 months postoperatively is much better after the application of collagen [43,44]. Collagen membranes are also very useful for periodontal procedures of guided tissue regeneration [45,46]. Collagen is characterized by biocompatibility as well as low immunogenicity and cytotoxicity. Taking into account the low immunogenicity of the polymer, the biomaterials obtained on its basis promote the growth of a native extracellular matrix, are chemotactic for fibroblasts and induce a cascade of events as a result of further development of the internal matrix. Collagen also has a strong osteoinductive effect, stimulating the differentiation and proliferation of osteoblasts, which leads to bone growth. It also increases gene expression for morphogenetic proteins and acts as an anchor for the attachment of proteoglycans and glycosaminoglycans, improving the overall mechanical strength and stability of the regenerated tissue. The inductive role of collagen in the expression of bone morphogenetic proteins influences the regeneration of peri-implant and periodontal damage [47].

#### 3.2.2. Fibrin

Fibrinogen—a glycoprotein present in human blood plasma—may be converted to insoluble clots of fibrin by the action of the serine protease thrombin. Due to its unique biological and physical characteristics, including porosity, deformability, elasticity and biodegradability, fibrin has great potential for application as a scaffold in tissue engineering [48]. A fibrin matrix with entrapped cytokines, growth factors and cells, called platelet-rich fibrin (PRF), is a very promising biomaterial that could be used in oral and maxillofacial surgery to improve bone healing in implant dentistry [49]. It is fully natural and biocompatible. It was proven that PRF enhances new bone formation and bone regeneration, and could help to reduce pocket depth [50].

#### 3.2.3. Gelatin

Another biocompatible polymer with adhesive properties and low immunogenicity, widely used in dental applications, is based on gelatin [51]. It is hydrolyzed collagen investigated for application in tissue engineering [52]. Drug forms prepared with the use of gelatin have high hemostatic activity and significantly improve the process of healing/regenerating damaged tissues, including post-extraction wounds [51,53,54]. As a polymer that promotes cellular attachment and growth but suffers from poor mechanical and antimicrobial properties, it could be used to create ideal wound dressing only after crosslinking with other polymers and the incorporation of antimicrobial agents [55].

#### 3.2.4. Bone Morphogenetic Proteins (BMPs)

Bone morphogenetic proteins (BMPs) are signaling molecules that belong to the transforming growth factor beta (TGF-β) superfamily. They are obtained from non-mineralized bone matrix. Since alterations in the activity of BMPs may be caused by various human diseases, the therapeutic application of BMP activators and inhibitors is a very promising approach [56]. Due to the ability to induce bone formation, BMPs play an important role in regeneration and bone remodeling [57]. They also increase bone response to alloplastic materials [58].

### 3.3. Polynucleotides

Polynucleotides are biopolymers composed of nucleotide monomers. The most common examples with distinct biological functions are DNA (deoxyribonucleic acid) and RNA (ribonucleic acid). In living organisms, polynucleotides encode the genome. Gene therapy is the future of contemporary medicine and will probably soon find an application in dentistry [59].

One of the most interesting therapeutic targets is cathepsin K (Ctsk), a lysosomal cysteine protease that is responsible for bone resorption, also possibly leading to bone destruction. Therefore, Ctsk-inhibitors are being sought to stop the possible destructive process in the periodontium, which manifests clinically as periodontitis. The inhibition of Ctsk may also be important in the prevention of temporomandibular joint diseases, as this factor causes bone resorption in joints as well [60].

Cancer treatment is a major challenge in medicine. It has been shown that the inhibition of tumor growth may be achieved with the use of graphene oxide-polyethyleneimine. The inhibitor complex in this pattern causes a decrease in the invasion and migration of oral squamous cell carcinoma. This also leads to the increased apoptosis of cancer cells, which significantly reduces tumor growth [61]. Reduced tumor growth could also be achieved with peptide-based cancer immunotherapy and antisense oligonucleotides. They reduce the expression of survivin, the factor responsible for the growth of tumors and their resistance to treatment. Downregulation of survivin expression may play an important role in the treatment of all types of tumors in the future. Furthermore, antisense oligonucleotides increase the apoptotic rate and make the tumor more sensitive to radiation and chemotherapy [62].

Another therapeutic option is based on oligonucleotide aptamers, which are becoming more popular in the treatment of microbial infections, as there are a lot of bacteria resistant to antibiotics. Single-stranded nucleic acids (DNA or RNA) are to replace monoclonal antibodies. They exhibit anti-biofilm and antimicrobial properties, as well as reduce the invasion of pathogens to immune cells. Oligonucleotide aptamers inhibit or reduce the effect of bacterial toxins during infection. Due to the high effectiveness of this kind of treatment, aptamers seem to be a very promising perspective in treatment, though targeted aptamers should be sought to support the treatment and diagnosis of specific tumors. Additionally, they might also be used in drug delivery systems [63,64].

## 4. Oral Applications of Natural Polymers

**Figure 3:**
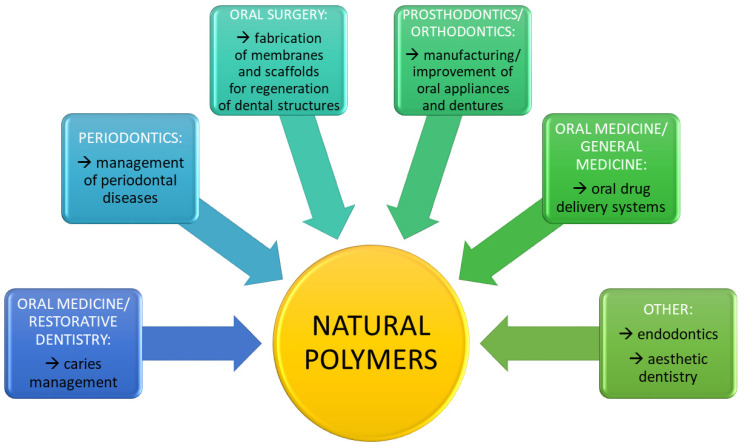
Summary of the applications of natural polymers in different dental specialties.

A general overview of the applications of natural polymers in various dental specialties is presented in Figure 3.

### 4.1. Caries Management

About two-thirds of the worldwide population suffer from caries. Due to high aesthetics and good physical and chemical properties, composites replaced amalgam restorations in the 1960s. It was proven that the currently used composite materials are not cytotoxic. Moreover, composites are more shapeable than amalgam fillings and, therefore, are more precise for dental restorations. Dental composites consist of a polymer matrix and an inorganic/organic filler. Considering natural polymers, chitosan-based organic–inorganic composite materials are used for the repair of dentine and enamel. They are produced to mimic the mechanical and biological properties of natural hard tissues. Nevertheless, those materials are heterogeneous and poorly repetitive. The particles aggregate unevenly, and there is no interaction between the organic and inorganic matrixes [65]. Chitosan could also be added to glass ionomer cements, which are another type of restorative material. Binaljadem et al. revealed that the addition of bioglass, processed bovine dentine and chitosan may increase cell viability [66].

Another important field of application for polymers is tissue engineering scaffolds used to regenerate the dentine-pulp complex. Importantly, this intervention enables avoiding root canal treatment, as an endodontically treated tooth is weaker and more prone to fractures [67]. It was proven that chitosan could reduce dentine loss when used in the treatment procedure [68]. Moreover, chitosan–collagen scaffolds have an odontogenic effect and may prevent endodontic treatment due to dentine tissue production [69]. Alginates could also be used as hydrogels aimed to form a scaffold for dentine or pulp regeneration. Among all the biomaterials, fibrin seems to have the best properties to restore pulp and reactionary dentin [67,70].

### 4.2. Periodontal and Oral Mucosa Disease Management

As periodontal diseases destroy periodontium, they may cause tooth loss. Barrier membranes, bone grafts and growth factors, or a combination of these, may be used to prevent further destruction and regain the bone and periodontium level. The present and the future of bone and periodontium reconstruction is based on the use of multiple materials, usually in combination. The biomaterials for tissue engineering improved the quality and results of periodontal procedures. For this purpose, both ceramics and polymers are used. Ceramic-based materials include calcium sulfate, calcium phosphate (hydroxyapatite, tricalcium phosphate) and bioactive glass. The most commonly used polymers are polysaccharides (e.g., chitosan) and polypeptides (collagen and gelatin) [71].

The perfect regeneration of periodontium includes the development of new cementum with periodontal ligament (PDL) fibers that are oriented perpendicularly to both the alveolar bone and the cementum [71]. A xenogeneic collagen matrix for soft tissue augmentation is the most recent invention. It acts similarly to the human bone and omits the ethical aspect of obtaining tissue from a donor [72]. Cementum and alveolar bone regeneration can also be achieved with the use of fibrin-ACP (fibrin enriched in chitosan particles releasing ε-aminocaproic acid (ACA)). The reconstruction of the periodontium in this method is based on the ability to promote cementogenesis with structural integration by Sharpey’s fibers [40]. Biopolymers could also be used to form scaffolds inserted in the periodontal pocket in order to restore tissue integrity and to treat bacterial infections. Budai-Szucs et al. proposed nanofibrous electrospun scaffolds based on gelatin and low molecular weight chitosan as a low-cost and easy to produce system providing support for the adhesion and proliferation of both fibroblasts and osteoblasts. On the other hand, such scaffolds could effectively reduce the proliferation of pathogens involved in periodontitis [73,74]. Another interesting observation was described by Chang et al., who proved that the addition of chitosan to antibiotics used for eradication of bacteria during the treatment of periodontitis not only strengthens the reaction against microbes but also prevents bone loss [75]. Due to high healing properties, hyaluronic acid is also used in topical administration as well as in coadjutant treatment for chronic and acute gingival and dental diseases. It may also be used for aesthetics to increase the lip volume (facial features) and restore gingival papillae when they are damaged [76,77]. On the other hand, the scaffolds based on HA were considered useless in pulp and dentine regeneration due to their poor mechanical properties and rapid degradation [67].

### 4.3. Manufacturing of Oral Appliances and Dentures

Dental prosthetics and orthodontics benefit from the broad spectrum of polymeric materials. However, thus far, mostly synthetic polymers are used for the fabrication of both partial and full dentures, bleaching trays, occlusal splints for temporomandibular joint treatment, mouth guards and retainers. Due to the complex chain structure of the material, polymeric splints have the ability to transform the energy from the deformation of the splint to vibration suppression. That prevents the oral cavity and teeth from injuries [78]. The major disadvantages of the most commonly used material, poly(methyl methacrylate) (PMMA), are its rather poor antimicrobial properties [79]. For this reason, the most important role of natural polymers in dental prosthetics and orthodontics is connected with an attempt to reduce microbial contamination of various oral appliances. Modification of PMMA with chitosan was proposed by Walczak et al. in order to achieve an antifungal effect. However, the results obtained are not satisfactory and require further investigation [80].

Clear overlay appliances (COAs) are a recent trend in orthodontics. The most popular methods of these are Invisalign^®^ and ClearAligner^®^. These splints, based on poly(ethylene terephthalate)-glycol (PETG), are a good alternative for classic brackets, especially due to their high esthetic value. The only disadvantages of this type of appliances are their low durability and weak antibacterial properties. To eliminate these drawbacks, multilayer films composed of chitosan and carboxymethyl cellulose were proposed as a system enabling the formation of a porous and rough film. The purpose of those layers was to prevent the adhesion of bacteria and to improve the mechanical resistance and stability of PETG. It was possible due to the increase in the bond strength between the polysaccharide chains [81].

Finally, the antifungal properties of natural polymers could be used in prosthetics and periodontology to cure *Candida albicans* stomatitis, which is the most common infection affecting prosthodontic patients [82].

### 4.4. Oral Tissue Regeneration and Oral Surgery

Dental implants are becoming the most common method for the restoration of lost teeth. Nevertheless, bone loss is an unavoidable phenomenon accompanying implantation. Bone grafts are the best and natural way of replacing bone, but due to moral considerations, people search for alternative methods. Substitutes include metals (tantalum, titanium, iron, magnesium), polymers (polylactides, polyglycolides, polycaprolactones, polyurethanes) and ceramics (silicates, calcium phosphates, calcium sulfate hemihydrate (CSH), calcium sulfate dihydrate (CSD), also known as gypsum) [83]. Natural polymers are also proposed as valuable materials with the potential of being applied in tissue engineering.

The ideal biomaterial for tissue engineering is biocompatible and biodegradable in a way corresponding to the rate of the new tissue restoration and is characterized by optimal mechanical properties and adequate morphology and porosity as well, simulating the lost tissue. It should act as a “bridge” between the polymer scaffold and the tissue environment, mimicking cells, metabolites, nutrients, gases and signal molecules. The main problem in tissue engineering is to grow the engineered tissue and maintain its vitality [43]. Natural polymers make it more feasible since providing oxygen and nutrition and rebuilding the vascular systems between the new cell formation are a *sine qua non* conditions for a modern biomaterial.

In dentistry, chitosan, collagen, hyaluronic acid, alginate and albumin could be used to substitute an extracellular matrix of skeletal muscle, bone or periodontium [19,84]. It was demonstrated that a scaffold coated with a gelatin layer and filled with chitosan nanoparticles loaded with bone morphogenetic protein 2 and dispersed into collagen hydrogel could serve as a biologically compatible framework for bone tissue engineering. The construct described above was an osteoinductive graft capable of the sustained release of BMP2 [85].

The healing of the alveolar socket before transferring the dental implant could also be enhanced with the use of natural polymers. For that purpose, a 0.2% HA gel might be used after surgery to help with healing or to cure oral ulcerations [30,86]. It is also considered helpful in treating alveolar osteitis after an extraction [87]. Moreover, some studies confirmed that a matrix based on hyaluronic acid mixed with autologous bone accelerates bone formation and its reconstruction in post-extraction sites [88]. On the other hand, Lopez et al. claim that there was no significant difference in wound healing regardless of whether or not hyaluronic acid was used [89].

Another natural polymer useful in oral surgery is collagen. Collagen membranes could improve bone regeneration and restoration. It has been shown that epithelial cells grow on collagen membranes just after 24 hours and form a structure similar to tissue after 48 hours [90,91,92]. Collagen is also used for cartilage restoration in temporomandibular disorders [93]. When degenerative changes occur, an effective regeneration of the temporomandibular joint (TMJ) is required. For this purpose, in some cases, surgical intervention is needed. In less complex cases, hyaluronic acid (HA) is used to provide an ideal chondrogenic microenvironment for cartilage regeneration [94,95,96]. HA promotes the synthesis of a cartilaginous matrix by the differentiation of stem cells into chondrocytes. This process supports cartilage regeneration [93,97,98]. Other natural polymers used for cartilage regeneration are agarose, fibrine, collagen and gelatin [95]. Gelatin could also be used for bone formation; it was revealed that the addition of 10% gelatin to the composite makes the structure of the graft more resistant to fracture [99]. Promising porous hydroxyapatite scaffolds for bone replacement could also be fabricated using gum tragacanth (GT) and other natural polymers as a binder [100,101].

### 4.5. Oral Drug Delivery Systems

The use of medical preparations in the oral cavity is a well-known method of treatment for local inflammation, pain and also diseases concerning the mucosa and dentition. The entire oral cavity is covered with relatively smooth mucosa. This determines the technological conditions for the development of preparations using polymers of natural origin.

In dentistry, the optimal way to use and fully understand the role of polymers and their impact on the modification of the interaction of medicinal substances with the site of application in oral cavity diseases is still being sought [102]. The ability of polymers to protect a fragile load makes them a perfect carrier used to reach the cellular target. Additionally, other aspects that should be considered are the issues of an enzymatic degradation of active pharmaceutical ingredients (API), objectionable taste, a low local penetration of the active substance and a fast elimination of the preparation from the application site [103].

The most popular polymer of natural origin used for the preparation of drug delivery systems is chitosan. An injectable chitosan sponge (guanosine 5′-diphosphate (GDP)) might be used for this purpose since it accelerates biomineralization in critical size bone defects and is cheap when compared to growth factors, e.g., BMPs [104,105]. Another material, particularly useful for antibiotic spread, is bacterial cellulose [106].

Due to the specific anatomy, histology and prevailing dynamics in the oral cavity, polymer carriers, as multifunctional pharmaceutical excipients, should be characterized by specific features related to the persistence of the drug at the site of its administration, e.g., mucoadhesive properties, gradual swelling, a modified release of the active substance, a protective effect against mechanical stimulation and easy application [107,108,109]. To achieve these properties in clinical conditions, in addition to conventional forms, i.e., rinses, mucoadhesive tablets or gels, the use of active substance carriers in the form of modified formulations is proposed. Among novel types of formulation—different films, fibers, biodegradable matrices, in situ gels or microspheres—nanoparticles or similar technological solutions should be mentioned [110,111]. This section provides examples and shows the benefits of developing technological solutions for the active delivery of substances designed on the basis of natural polymers for application as local therapies for lesions in the oral cavity.

Conventional semi-solid gel formulations are used for the application of active substances, most often with antibacterial, anti-inflammatory and aseptic properties. This allows their non-problematic application by the dentist or by the patient while ensuring the relatively quick release of the drug at the site of application. Their main advantage is a low risk of irritation [112], as well as good rheological properties and good adhesion to differentiated oral mucosa [113].

A modification of classic gel formulations resulted in the introduction of sol-gel formulations into therapy. They are obtained by using polymers sensitive to factors such as temperature or pH change. While these change, the system begins to gel in situ at the application site, extending the time of the formulation remaining at the site of application and, as a result, prolonging the release of the administered active substance. The natural polymers sensitive to such factors include, i.e., chitosan and its derivatives, alginates, cellulose, gellan gum and gelatin [114,115,116,117].

Other formulations developed for use in the oral cavity are solid carriers, i.e., films, matrices and polymer nanofibers obtained based on cross-linked gelatin, collagen or hyaluronic acid [118,119,120]. The most important advantages of such systems are their mucoadhesion and mechanical properties (e.g., high strength and resistance to quick diffusion, as well as flexibility, allowing their easy application to the periodontal pocket). The favorable cross-linking of gelatin carriers affects their swelling capacity, biodegradation and the release of substances from the developed matrices [121,122].

Different multi-compartment preparations such as polymer-coated liposomes, polymer nanoparticles or microspheres have been also proposed for the treatment of oral diseases [123,124,125]. They often are characterized by bioadhesive properties or responsiveness to stimuli in targeted therapy for oral cancer. They may also be used in addition to photodynamic therapy for the delivery of photosensitizers in photodynamic therapy [125,126]. The possibility of the selective destruction of neoplastic cells with the simultaneous reduction in cytotoxicity toward non-cancerous tissues, as well as bactericidal activity against bacteria forming oral biofilm, are additional benefits of the use of nanocarriers in oral cancer therapies [127].

The examples of active ingredient carriers based on natural polymers used for local applications in various therapies for oral cavity diseases are presented in Table 1.

### 4.6. Other Uses

The use of osteoplastic materials in orofacial medicine resulted in considering their use for the rebuilding of skull bone, i.e., cranioplasty. The main types of materials used for that purpose are hydrogels and cements. The most popular natural polymers used for the production of hydrogels are collagen, alginate, chitosan, gelatin, hyaluronic acid, cellulose and fibroin [135]. Hydrogels are safe and bioresorbable but, compared to cements, their mechanical properties are not satisfactory and need improvement. Bone cements are composed of a polymer powder and a liquid monomer. The main two types of cement are polymethyl methacrylate (PMMA) and calcium-phosphate cement (CPC). Unfortunately, bone cements are not resorbable and do not induce osteogenesis. They also do not adhere to bone. The last problem with this group of polymers is that the reaction of polymerization is exothermic [136].

Another dental specialty that may benefit from natural polymers is endodontics. Chitosan was the most intensively investigated in terms of application in this field. It might be used in root treatment as an irrigating solution. The potential effectiveness against specific microorganisms (especially those resistant to antibiotics, such as *E. faecalis* and *C. albicans*), as well as its biocompatibility with the periapical tissues, make chitosan a useful alternative natural substance for endodontic treatment. It can also be applied for direct and indirect pulp capping [78,137]. Additionally, it may also be used as a chelator, able to remove debris occurring during endodontic treatment or calcium hydroxide medicament during endodontic treatment. It was suggested that chitosan is more effective in the removal of topically delivered drugs during endodontic procedures when it is applied in combination with ultrasounds [138]. Apart from endodontics, chitosan might also be applied in conservative dentistry, as an ingredient of toothpaste with antibacterial properties, enabling the prevention of periodontal inflammatory processes [104].

Last but not least, aesthetic dentistry could also make use of the wealth of nature. Hyaluronic acid is commonly used in esthetic medicine for soft tissue augmentation, to improve skin hydration and stimulate collagen and elastin production. It also restores face volume and has rejuvenating properties, acting as a “space-filler”. Therefore, it is a perfect solution to complete the effects of orthodontic or ortho-prosthodontic therapy [139].

## 5. Natural vs. Synthetic Polymers for Dental Application

**Figure 4:**
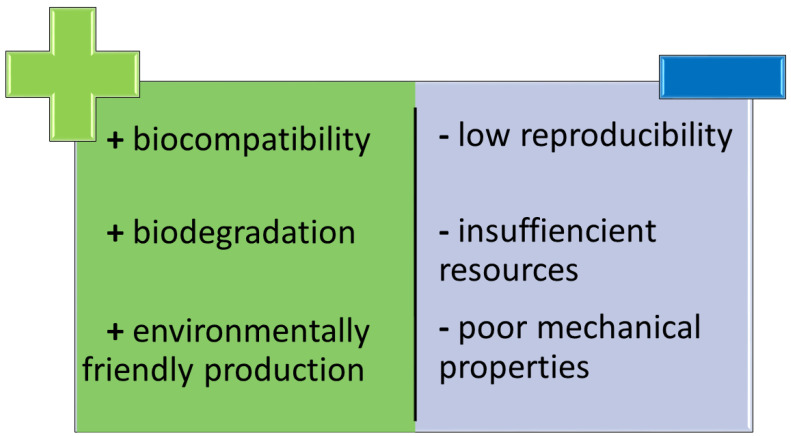
Comparison between advantages(+) and disadvantages(−) of polymers originated from natural sources.

The development of dental biomaterials has significantly improved treatment options and their results. Synthetic polymers, including, i.e., polylactic acid (PLA), polyglycolic acid (PGA) and polylactide-co-glycolide (PLGA), are cheap and reproducible [67]. Not all of them are degradable, unlike polymers of natural origin [8,140]. The main advantage of natural polymers is their biocompatibility, low immunogenic response and the lack of adverse effects of its production on the environment and humans. On the other hand, their resources could be insufficient in relation to the demand. Another problem may also be connected with poor mechanical strength and resistance [141,142] (Figure 4).

Even though natural polymers demonstrate good cell biocompatibility, i.e., the ability to support the survival and functioning of cells when selecting them for use in dental practice, attention should be paid to the problem of the variability of their source. Another important issue concerns immunogenicity and the often, in contrast to synthetic materials, limited range of possibilities of changing their mechanical properties or controlling the pore size of the materials obtained on their basis [143]. The low mechanical strength and high degradation rate of natural polymers often require their use in combination with other semi-synthetic or synthetic polymers, or ceramics, obtained in cross-linking reactions to reduce the rate of degradation. The addition of synthetic polymers allows the materials to be tailored to meet absorption time requirements, potentially facilitating repeatability and upscaling [74]. Detailed information concerning the disadvantages and possible risks posed by natural polymers, both in general and individually, is presented in Table 2.

## 6. Perspectives

The population is aging, and looking for new methods of treatment and healthcare prevention that are both biologically neutral and effective for the elderly is one of the main challenges of the 21st century. Thanks to technological development, living according to the maxim “forever young” could become a reality. Dentistry also follows this trend.

One of the most important factors enabling the treatment, improvement or even replacement of oral tissues, is biomaterials. Understanding the biology and physiology of the human body more and more, it becomes clear that since polymers are a natural part of it, their replacement should be sought in nature. Novel perspectives offer not only a possibility of maintaining oral health, but also regaining it and reversing the process of hard tissue loss. It was suggested that the addition of chitosan to Ca^2+^ ions enables the production of scaffolds used for the reconstruction of dental hard tissues. Chitosan makes those structures more porous and bioactive, forming a perfect skeleton for new dentine [67,153]. Biomaterials may also be used for wound healing as they help in the proliferation of stem cells [154].

A particularly interesting technology providing a novel use of biomaterials is 3D bioprinting [155,156]. Natural products are used in bioprinting most commonly in the form of hydrogels. They do not need organic solvents or high temperatures, which makes them a perfect component for printing living tissues. The main requirements for these materials include printability, tissue biomimicry and appropriate mechanical properties, as well as biocompatibility and biodegradability. These features of microgels play an important role in cell adhesion and interaction, resulting in spheroid formation [157,158,159].

With the development of 3D bioprinting, the utility of polymers and composites has increased. This technology promotes the development of personalized medicine, allowing the manufacturing of products that are patient-specific and anatomically matched. Fused deposition modeling (FDM) is the most common technique, where a thermoplastic polymer is melted and then extruded on another layer of it or on a special platform. The printed structure solidifies at room temperature. Due to the high temperatures used in that process, all the cells and growth factors are applied after the production of the print to avoid the inactivation of the growth factors or even cell death. Although this technique has been known for decades, the past few years showed that 3D printing is the future of all areas of life, including dentistry, since it allows for the production of all types of dental products customized for the individual patient.

**Figure 5:**
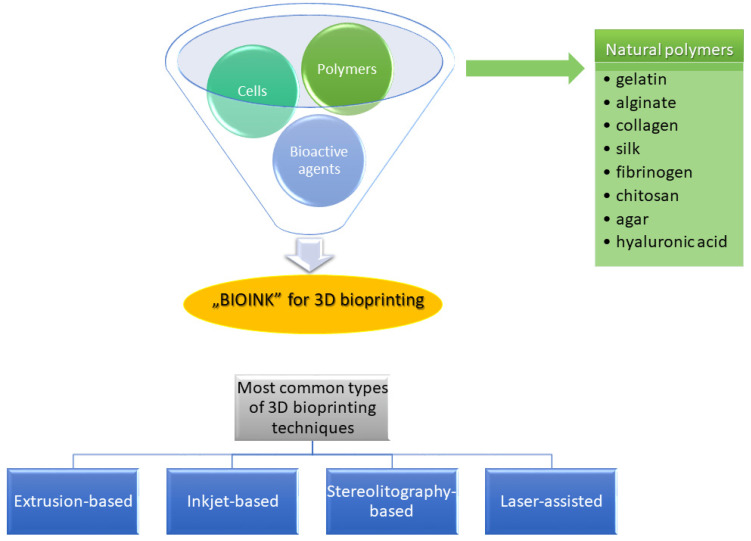
Summary of 3D bioprinting technology: examples of natural polymers used for a fabrication of “bioinks” and the most common types of bioprinting techniques [160,161].

The most common types of natural polymers used as components of “bioinks” in various 3D bioprinting technologies include gelatin, alginate, collagen, silk, hyaluronan, chitosan, fibrinogen, agar and decellularized extracellular matrix (dECM) (Figure 5). Commercially available bioinks based on polymers of natural origin were described by Liu et al. [160]. They can be used in various 3D bioprinting techniques, including extrusion-based bioprinting, inkjet bioprinting, stereolithography-based bioprinting and laser-assisted bioprinting. The advantages and limitations of each of these procedures were compared by Khoeini et al. [161].

## 7. Study’s Limitations

Due to the exclusion of non-English language papers, some valid data in other languages may have been missed. Additionally, as materials science constantly evolves at a fast rate, and the fact that natural methods of treatment still attract the attention of many research groups, this review could not discuss the issue comprehensively enough. On the other hand, apart from presenting the current applications of natural polymers in dentistry, it also indicates the most promising perspectives for these materials.

## 8. Conclusions

The development of new strategies of both oral health maintenance and oral diseases treatment is strongly related to advancement in a field of biomaterials. For this reason, searching for novel solutions attracts the attention of many research groups. The current review could be a “bridge” between material scientists and dentists, since it offers a critical and comprehensive summary of the most up-to-date findings regarding the dental applications of polymers of natural origin. It highlights the specific requirements that must be taken into account when proposing new materials potentially attractive for application in the increasingly demanding field of modern dentistry. On the other hand, this review indicates future perspectives and should inspire doctors to look for further information on the novel, innovative systems that could be used to improve the quality of life of their patients.

The highly desirable properties of natural polymers, such as availability, the capability of chemical modifications, biodegradability and biocompatibility, make them very attractive materials. They could be preferably used in almost every field of dentistry, including caries management, periodontology, prosthodontics and the regeneration and reconstruction of oral tissues. They are also very useful components of rationally designed drug delivery systems. The versatility of their characteristics allows them to be even more frequently adapted in other fields of medicine in the future. New applications for these promising materials are still being developed, including for gene therapy.

On the other hand, the major disadvantages of natural biomaterials, related to unsatisfactory mechanical properties, considerable variability and immunogenicity, significantly limit their potential. Taking into account the existing limitations of the current materials, a wide range of naturally derived biopolymers is continually being developed to meet the increasing requirements of modern dentistry. Recent progress in materials science allowed for the modification of natural polymers aimed at enhancing their biofunctionality. As a result, some novel properties tailored for specific applications could be ensured. In particular, different methods of improving mechanical properties, inducing a desired biological response (e.g., cell adhesion or proliferation) and creation of “smart”, stimuli-responsive polymers have been proposed. Owing to their functionalization, novel materials with adjusted, desired properties could be obtained to better meet the growing expectations of modern dentistry.

## Figures and Tables

**Table 1:** The examples of active pharmaceutical ingredient (API) carriers based on natural polymers, used for local applications in therapies for oral cavity diseases.

Polymer	API	Effect	Ref.
chitosan and its derivatives	chlorhexidine	Antimicrobial, biodegradable and thermosensitive carrier, releasing chlorhexidine in a prolonged manner, potentially useful in the treatment of periodontal diseases.	[114]
	metronidazole	The synergistic activity of the polymer and substances in the form of hydrogel, used in the treatment of chronic periodontitis.	[128]
	metronidazole and levofloxacin	Films based on cross-linked chitosan provide a long-term release of the substance for up to 7 days. Clinical trials confirmed the therapeutic efficacy of the films in reducing the clinical indicators of periodontitis, i.e., gingival index, plaque index and periodontal pocket depth.	[118]
	clindamycin phosphate	Microparticles for local drug delivery to the periodontal pocket for sustained release of the antimicrobial drug; promising in the treatment of periodontal diseases.	[124]
	celecoxib	Mucoadhesive chitosan gels with laurocapram as non-ionic surfactant considered as a potential system for buccal administration of celecoxib in the chemoprevention of oral cancer.	[129]
	5-aminolevulinic acid (ALA)	Succinate-modified chitosan (SCHI) nanoparticles complexed with folic acid, forming nanostructures with high ALA incorporation efficiency and improving ALA release in cell lysosomes. The obtained nanoparticles are an excellent vector for the specific oral delivery of 5-ALA for the photodynamic detection of oral cavity cancer cells.	[125]
	atorvastatin	Chitosan-based mucoadhesive gels containing 2% atorvastatin were evaluated in periodontitis rats. There was a reduction in the release of the pro-inflammatory cytokines IL-1β, IL-6 and IL-8 and the anti-inflammatory cytokines TGF-β1 and TGF-β2, with significant healing of the alveolar bone, comparing to the control group. The anti-inflammatory effect was increased when atorvastatin was delivered using chitosan-based gel. The developed gels are potential carriers for substances from the group of statins in the treatment of periodontitis.	[130]
	lidocaine hydrochloride	The anesthetic effect of mucoadhesive hydrogels based on chitosan glutamate was evaluated in vivo after application to the buccal mucosa, in comparison with commercial semi-solid formulations containing lidocaine hydrochloride. The obtained mucoadhesive hydrogels may be useful in reducing pain symptoms in aphthosis and other painful lesions of the oral mucosa.	[131]
gellan gum	moxifloxacin hydrochloride	In situ gelling carriers were prepared based on ion-sensitive gellan gum and temperature-sensitive Poloxamer 407, finally to obtain prolonged retention at the application site. The optimized formulation with 19.072% Poloxamer 407 and 0.245% gellan gum has shown appropriate properties as an in situ carrier for drug delivery in dental application.	[115]
karaya gum/badam gum	moxifloxacin hydrochloride	Gels based on natural polymers (karaya gum (1, 2, 3%), badam gum (1, 2, 3%) and chitosan) with moxifloxacin hydrochloride showed potential for their application in the treatment of periodontitis.	[132]
gum extracted from ripe dillenia fruit	aceclofenac	Toothpastes containing 1% aceclofenac and dillenia fruit gum had beneficial rheological properties for better extrusion from the tube and spread. The in vitro release of aceclofenac from dental pastes was sustained for 6 hours. The pastes showed excellent mucoadhesion to the mucosa of porcine cheek, which may be beneficial for applications in the treatment of pain and periodontitis.	[133]
hyaluronic acid	-	Gels containing 0.8% hyaluronic acid were proposed for subgingival application as an adjunct for scaling and root planing (SRP) in the treatment of generalized chronic periodontitis. The statistically significant reduction in periodontal pocket depth, as well as the increase in the relative attachment level (RAL), were observed when compared to the control group.	[134]
pectin	-	Pectin-coated liposomes were described as promising oral drug carriers, able to adsorb onto hydroxyapatite as a model substance for human tooth enamel.	[123]
alginate/derivatives	metformin hydrochloride	Mucoadhesive multilayer film containing metformin hydrochloride with osteogenic activity was developed for intrapocket application in the treatment of periodontitis. The inner layer containing the drug was formed by carboxymethyl cellulose sodium salt (CMC) or sodium alginate. Thiolated sodium alginate (TSA) (2 or 4%) was the drug-free outer layer used to increase mucoadhesion and to achieve controlled release of the substance. A film based on CMC in the outer layer with 4% TSA with enhanced mucoadhesion and in vitro controlled drug release (83.73% within 12 h) was clinically evaluated in 20 patients. Clinical trials revealed improvement in all clinical parameters after six months of treatment, suggesting that the local use of mucoadhesive multilayer films with metformin hydrochloride may become an effective treatment method for moderate chronic periodontitis.	[119]
gelatin	ethanolic propolis extract	Formulations based on 1% gelatin revealed thermosensitive properties (Tsol/gel), appropriate for the oral mucosa application. Modified kinetics of in vitro release of substances from gelling formulations, inconsistent with Fick’s law of diffusion, was achieved.	[116]
	nystatin	Gelatin-based electrospun nanofibrous scaffolds for sustained drug release in the mouth were prepared. To improve the structural stability of the scaffolds and to enable the non-invasive incorporation of substances, photoreactive polyethylene glycol diacrylate was used as a cross-linking agent stabilizing by forming semi-interpenetrating network gelatin nanofiber scaffolds. The cross-linked structures effectively increased the structural stability of the obtained nystatin scaffold with statin in aqueous solutions.	[122]
	curcumin	The cross-linked gelatin film released the substance in vitro over a period of up to 7 days. The carrier may be potentially used for topical drug delivery to periodontal pockets.	[121]
collagen	chloramphenicol	Collagen sponges cross-linked with glutaraldehyde with chloramphenicol showed resistance to degradation by collagenase and strong antibacterial activity. Considering requirements for oral cavity drug carriers, i.e., low water absorption, slow drug release, high drug loading, high antimicrobial activity and resistance to enzymatic activity, optimal properties were obtained for 0.5% glutaraldehyde content in a sponge. The properties of the obtained formulations showed that sponges may be useful in the treatment and/or prevention of infected oral cavity lesions.	[120]

**Table 2:** Summary of disadvantages and possible risks posed by natural polymers.

Polymer	Disadvantages and Risk of Use as a Drug Carrier	Reference
Generally	Variability of the source of their origin Batch-to-batch variation resulting from various environmental and physical factors Presence of impurities, i.e., heavy metals, proteins, microbial contaminations Slow rate of production Immunogenicity Price Limited range of possibilities to differentiate their mechanical properties	[143]
Alginate derivatives	Multistage extraction and purification process, necessary to eliminate various types of impurities, including heavy metals, proteins, endotoxins and toxic polyphenols The degree of alginate biocompatibility depends on its composition, in particular the ratio of poly α-L-guluronic acid and β-D-mannuronic acid blocks, and the purity level	[144]
Chitosan and its derivatives	Various properties of materials obtained from various sources, both animal (exoskeleton of marine animals such as shrimp, crabs or lobsters) and non-animal (mushrooms, yeasts) Difficult and expensive to obtain and process	[145]
Cellulose derivatives	Properties determined by the type and degree of substitution, but also by the functionalization pattern along the polymer chain; derivatives may vary in terms of chemical structure, moisture sorption, water interaction, surface activity and solubility Potential pharmaceutical side effects and cytotoxicity profile of this material still need to be evaluated	[146]
Hyaluronic acid	Various types of contamination (viruses and animal proteins for HA extracted from animal sources, and toxins for HA extracted from bacteria) may trigger an immune response HA is often degraded quickly by hyaluronidases—for some medical applications the extracted HA must be chemically modified to extends residence time in the application site	[147]
Agarose	Low degradation rate is a limitation for in vivo application Due to the low ability to be loaded with higher concentration of hydrophobic drugs, there is a need for chemical modifications	[148]
Collagen	High cost of obtaining pure type of collagen I Variability in terms of fiber size, cross-link density, trace impurities Variability in enzymatic degradation rate as compared with hydrolytic degradation Side effect as a bovine spongiform encephalopathy (BSE) and mineralization Hydrophilicity that leads to swelling and more rapid release	[149,150]
Gelatin	Poor batch-to-batch reproducibility Animal-origin gelatins can pose risk of contamination with transmissible spongiform encephalopathies Due to a short degradation rate, poor mechanical properties and thermal instability of gelatin carriers, incorporation of various cross-linking agents is often required Compared with collagen, gelatin is highly susceptible to several proteases, which may lead to its faster degradation	[151]
Fibrin	High cost Poor batch-to-batch reproducibility Due to its rapid degradation and weak mechanical properties, combination with other materials is often required.	[152]
